# Overcoming Double Jeopardy: Successful Orthotopic Heart Transplant in a Recipient With Bacterial and Fungal Infections

**DOI:** 10.1155/2024/4175313

**Published:** 2024-07-17

**Authors:** Paopat Munthananuchat, Bundit Naratreekoon, Narongrit Kantathut, Piya Samankatiwat, Akeatit Trirattanapikul, Teerapat Yingchoncharoen

**Affiliations:** ^1^ Department of Medicine Faculty of Medicine Ramathibodi Hospital Mahidol University, Bangkok, Thailand; ^2^ Division of Cardiology Department of Medicine Faculty of Medicine Ramathibodi Hospital Mahidol University, Bangkok, Thailand; ^3^ Division of Cardiovascular Thoracic Surgery Department of Surgery Faculty of Medicine Ramathibodi Hospital Mahidol University, Bangkok, Thailand; ^4^ Division of Infectious Diseases Department of Medicine Faculty of Medicine Ramathibodi Hospital Mahidol University, Bangkok, Thailand

**Keywords:** cardiac assist devices, heart transplant, infection, invasive pulmonary aspergillosis

## Abstract

Although active infection is generally a contraindication before an orthotopic heart transplant, a 16-year-old man diagnosed with dilated cardiomyopathy successfully underwent an orthotopic heart transplant despite having active probable invasive pulmonary aspergillosis and bacterial pneumonia in the presence of septic and cardiogenic shock.

Summary

• To recognize the appropriate timing of orthotopic heart transplant in the setting of active infection.
• To understand the appropriate preoperative and postoperative management of invasive pulmonary aspergillosis in orthotopic heart transplant patient.

## 1. Introduction

Heart transplantation is considered an important treatment option for advanced heart failure patients who do not respond to medical therapy. However, before surgery, contraindications for heart transplantation should be evaluated. These contraindications may include fixed pulmonary hypertension, active cancer, HIV infection, or active infections with unstable conditions. [1]

Infection is a significant concern for patients undergoing heart transplantation, including the pre- and postoperative periods, as it may lead to the risk of severe infections in these patients. In fact, infections are the second leading cause of death after graft rejection within the first 30 days following heart transplantation and the leading cause of death beyond 30 days post-transplantation. [2, 3]

Bacterial infections are significant concern for patients undergoing heart transplantation. According to a study, the incidence of bacterial infections in these patients can be as high as 43.6%. Common causes of infection include pneumonia, bloodstream infections due to the placement of central venous catheters, and infections associated with the use of ventricular assist devices. In the past, the mortality rate from invasive pulmonary aspergillosis in heart transplant patients was as high as 66.7%. However, advancements in medical treatments, including antifungal medications, adjustment of immunosuppressive therapy, and improved surgical techniques, have improved survival in these patients. [4, 5]

As a result, current medical guidelines recommend treating fungal infections until symptoms, radiological findings, and laboratory tests show improvement before proceeding with heart transplantation. However, there are no specific recommendations for the optimal duration of treatment before emergency heart transplantation or in cases where the patient's condition requires urgent surgery. [6]

## 2. History of Presentation

A previously healthy 16-year-old man presented with gradually worsening dyspnea on exertion for over 5 months. As the dyspnea progressed, he had a low-grade fever for 2 days and was initially diagnosed with congestive heart failure and acute respiratory failure at the general hospital. Despite being treated with mechanical ventilator support for 4 days, the patient's condition did not improve, leading to the development of cardiogenic shock. In consequence, he was immediately transferred to the cardiac care unit (CCU) of Ramathibodi Hospital (Ramathibodi Hospital is a university hospital providing super tertiary care located in Bangkok city in the central region of Thailand) for further management.

On admission, the patient had a body temperature of 36.0°C with a heart rate of 130 beats per minute, an oxygen saturation level of 92%, and a blood pressure of 85/50 mmHg. Physical examination showed an engorged neck vein, fine crepitation in both lower lung zones, normal heart sounds without murmur, and cold extremities with delayed capillary refill. Initial transthoracic echocardiogram revealed left ventricular dilatation with an ejection fraction of 10%. Acute myocarditis was suspected, and the patient underwent the insertion of venoarterial extracorporeal membrane oxygenation (VA-ECMO) and intraaortic balloon pump (IABP) to stabilize hemodynamics. Furthermore, an atrial septostomy procedure was performed to alleviate the pressure on the left ventricle. After 4 days of intensive treatment with mechanical circulatory support, a decision to bridge to an orthotopic heart transplant was made.

**Figure 1:**
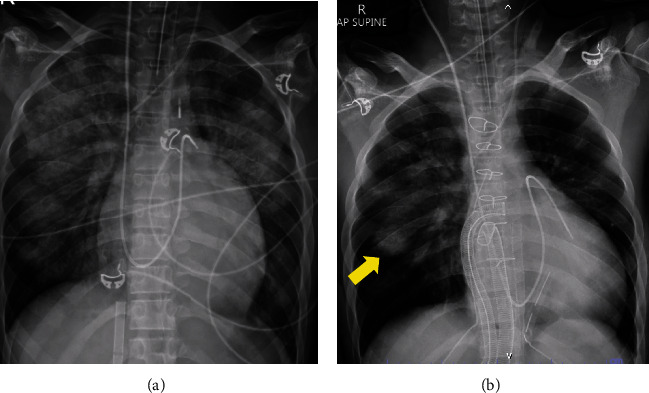
Comparison of chest X-ray findings on the 1^st^ day and 9^th^ day of admission. (a, b) On the 9^th^ day of admission, after adequate decongestion, the follow-up chest X-ray revealed persistent pulmonary infiltration right middle lung zone (yellow arrow).

**Figure 2:**
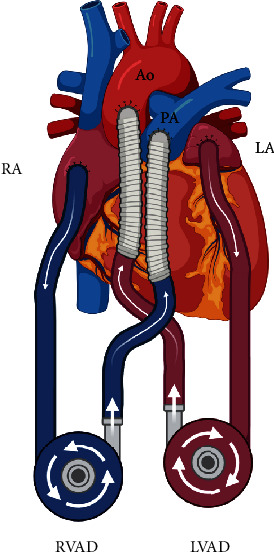
Biventricular assist device configuration. A visualization of a biventricular assist device (BiVAD) with a configuration where the left ventricular assist device (LVAD) inflow cannula is connected via the left atrium (LA); the outflow cannula is connected via the ascending aorta (Ao). The right ventricular assist device (RVAD) inflow cannula is connected via the right atrium (RA), and the outflow cannula is connected via the pulmonary artery (PA), created with BioRender.com.

Transthoracic echocardiogram after VA-ECMO placement revealed severe left ventricular dilation with left ventricular ejection fraction of 19% by biplane method, global wall hypokinesia, right ventricular systolic dysfunction, complete aortic valve closure, mild to moderate secondary mitral regurgitation, and other valves were not significant dysfunction. After adequate decongestion, a chest X-ray showed persistent pulmonary infiltration in the right middle lung zone (Figure 1). On the ninth day, the VA-ECMO was switched to a biventricular assist device (BiVAD) using the CentriMag circulatory support system with the surgical configuration shown in Figure 2. The patient was then listed for an urgent orthotopic heart transplant.

## 3. Past Medical History

The patient had no significant medical history.

## 4. Differential Diagnosis

Differential diagnosis of acute heart failure includes lymphocytic myocarditis, viral myocarditis, and decompensated dilated cardiomyopathy. Regarding the persistent infiltration observed in the right middle lung zone, various conditions can be considered in the differential diagnosis. These include localized pulmonary edema and pulmonary infections such as bacterial pneumonia, pulmonary tuberculosis, or fungal infection.

## 5. Investigations

**Figure 3:**
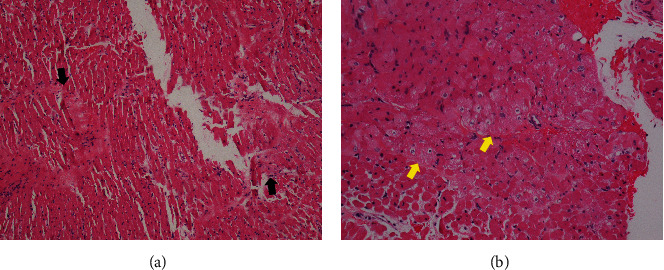
Pathological findings from endomyocardial biopsy on the 9^th^ day of admission. (a) The black arrows indicate mild interstitial fibrosis. (b) The yellow arrow indicates vacuolar degeneration.

A comprehensive workup of acute myocarditis conducted included viral infection, *Mycoplasma pneumoniae* infection, and toxin screening. All test results were negative. Additionally, an endomyocardial biopsy was performed, and the pathological findings revealed mild interstitial fibrosis and no pathological evidence of acute myocarditis (Figure 3).

**Figure 4:**
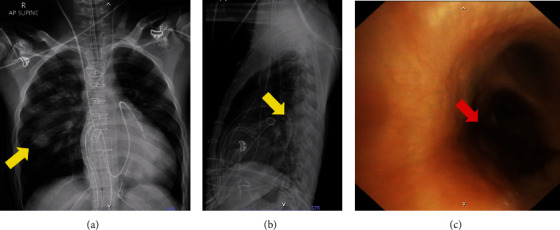
Plain AP and lateral chest X-ray and bronchoscopic finding. (a, b) Comparison of chest X-ray views indicating a suspected superior segment of right lower lobe lesion (yellow arrows). (c) Bronchoscopic findings reveal blood streak on bronchus intermedius (red arrow).

Further investigation for the persistent infiltration in the right middle lung zone was performed. A chest CT scan could not be performed due to the unstable condition and mechanical circulatory support. As an alternative, a lateral plain chest X-ray was done, which indicated a suspected lesion in the superior segment of the right lower lobe (RLL). The finding of bronchoalveolar lavage (BAL) through fiberoptic bronchoscopy was an old blood streak with mild mucosal swelling at the distal right bronchus intermedius (Figure 4). Separate BAL samples were collected from the superior segment and posterior segment of the RLL and were sent for aerobe culture, cryptococcal antigen, and *Aspergillus* galactomannan antigen. The aerobe culture of the BAL fluid showed *Trichosporon asahii* and trimethoprim-sulfamethoxazole-resistant *Stenotrophomonas maltophilia*. Additionally, the *Aspergillus* galactomannan antigen and cryptococcal antigen test were positive for BAL fluid from the superior segment of the RLL. Upon discussing the result of the BAL fluid cryptococcal antigen, an additional serum cryptococcal antigen test was performed which came back negative. This raised suspicion of a false positive cryptococcal antigen due to cross-reaction with *Trichosporon asahii* [7]. Considering the overall test results, the patient was diagnosed with probable invasive pulmonary aspergillosis (IPA) in conjunction with *Stenotrophomonas maltophilia* ventilator-associated pneumonia (VAP). Hemoculture was obtained, and the results returned negative for aerobic culture.

## 6. Management

During the initial diagnosis of cardiogenic shock, hemodynamic stability was maintained through using mechanical circulatory support. Medical management involved the administration of inotropic drugs. The antibiotics and antiviral prescribed included ceftriaxone, azithromycin, doxycycline, and oseltamivir to cover potential causes of myocarditis. After the VA-ECMO insertion, the dosage of inotropic drugs could be gradually reduced while still maintaining adequate hemodynamics. Hemodynamic parameters, mechanical circulatory support, and inotropic agents are summarized in Table 1.

On the 10^th^ day after BiVAD insertion, the patient's hemodynamics deteriorated even though BiVAD function remained normal, and there were no mechanical complications found on the echocardiogram. Higher vasopressor doses were required to maintain mean arterial pressure. Persistent pulmonary infiltration raised suspicion of VAP with septic shock. Empiric antibiotics were administered, including meropenem, vancomycin, levofloxacin, trimethoprim-sulfamethoxazole, and colistin. After the BAL results came back, antibiotics were changed to meropenem, tigecycline, levofloxacin, and voriconazole. Fortunately, after implementing intensive management, the patient's hemodynamics improved within 24 h. This positive response led to an urgent orthotopic heart transplant. However, due to active VAP with septic shock within the past 24 hours as well as the diagnosis of probable IPA, a lower immunosuppressive regimen was chosen. This regimen consisted of intravenous methylprednisolone 500 mg and oral mycophenolic acid 540 mg administered prior to the operation.

**Figure 5:**
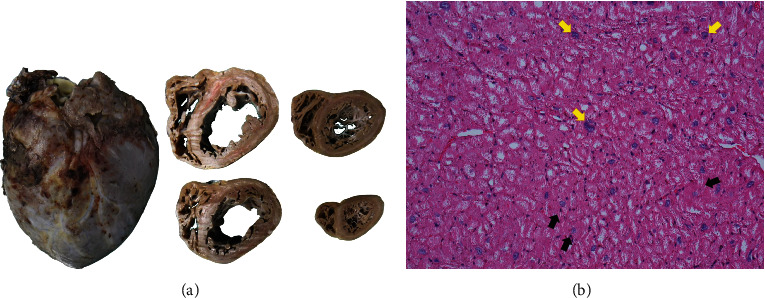
Pathology of the explanted heart. (a) Gross pathology showed biventricular dilatation with no significant ischemia or fibrosis that is consistent with dilated cardiomyopathy. (b) Microscopic findings revealed myocyte hypertrophy (yellow arrows) and vacuolar degeneration (black arrows).

After the 20^th^ day, the patient underwent an orthotopic heart transplant. The explanted heart's pathological examination revealed dilated cardiomyopathy without a specific etiology, leading to the diagnosis of idiopathic dilated cardiomyopathy (Figure 5).

Following the successful transplant, the overall clinical condition showed improvement, enabling the removal of all mechanical circulatory support and the discontinuation of antibiotics within 7 days. Voriconazole was continued to treat probable IPA, in addition to immunosuppressive drugs such as tacrolimus, mycophenolic acid, and prednisolone.

## 7. Discussion

Ramathibodi Hospital started its heart transplantation program in May 2017. Since then, over almost 7 years, the hospital has carried out a total of 21 heart transplant surgeries, plus one recardiac transplantation. Notably, the most recent procedure, conducted in February 2024, involved a combined heart and liver transplantation, indicative of the hospital's proficiency in complex surgical interventions with an average of approximately three cases per year.

IPA was a significant cause of morbidity and mortality among heart and other solid organ transplant recipients [4]. The active infection raised concerns about the transplant timing.

**Figure 6:**
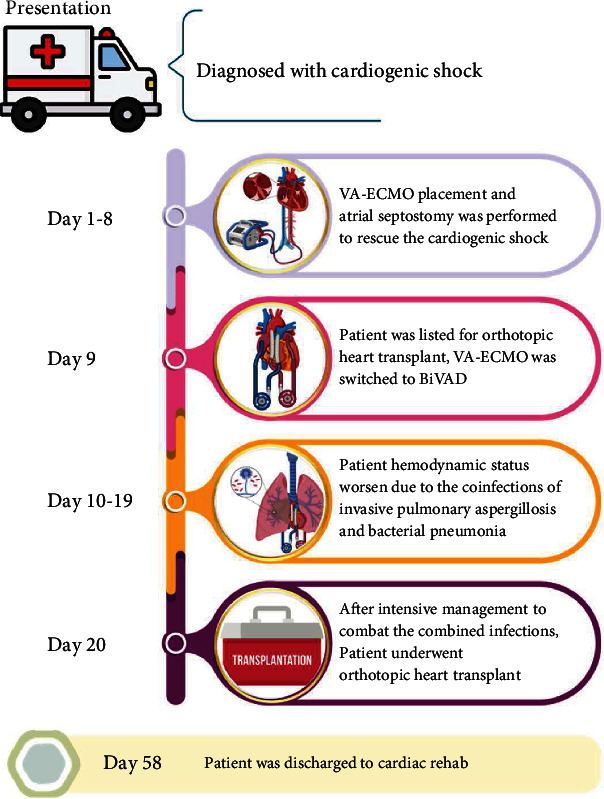
Timeline of admission, created with BioRender.com.

In this case, the timeline is demonstrated in Figure 6. The decision to proceed with an orthotopic heart transplant in the presence of an active infection required careful consideration by a multidisciplinary team. During the perioperative and postoperative periods, the team had to weigh the risks and benefits of the transplant simultaneously with an ongoing infection. The decision was made to minimize the risk of exacerbating the infection or compromising the patient's post-transplant outcome and the potential consequences of delaying the transplant.

## 8. Follow-Up

**Figure 7:**
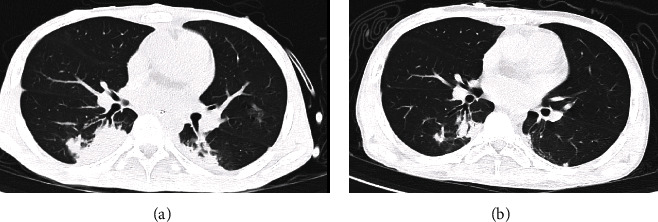
Comparison of computed tomography scan of the chest showed improvement in the 6^th^ week after voriconazole treatment. (a) Two weeks post-treatment: consolidation observed in both lower lobes, with greater prominence in the right lower lobe. (b) Six weeks post-treatment: significant resolution of consolidation in the right lower lobe.

The chest CT scans were performed in the 2^nd^ and 6^th^ week after initiating voriconazole treatment, which revealed a gradual improvement in the consolidation observed in the RLL (Figure 7). To monitor for rejection, serial endomyocardial biopsies were performed, and all pathological results did not show any significant concerns.

## 9. Conclusion

We demonstrated that controlled infection within a 24-hour period and the presence of probable IPA may not necessarily be a contraindication for orthotopic heart transplant. However, the decision should be carefully weighed on an individual basis and should not be extended to general practice.

## Figures and Tables

**Table 1:** Hemodynamic parameters, mechanical circulatory support, and inotropic agents during admission.

**Date/time**	**SaO2** **(%)**	**SvO2** **(%)**	**CO** **(L/min)**	**CI** **(L/min/m** ^**2**^ **)**	**MAP** **(mmHg)**	**CVP** **(mmHg)**	**PCWP** **(mmHg)**	**HR** **(bpm)**	**SVR** **(dynes**∗**sec/cm**^**-5**^**)**	**Lactate** **(mmol/L)**	**MCS**	**Norepinephrine** **(mcg/kg/min)**	**Milrinone** **(mcg/kg/min)**	**Procedure**
First day														
2.00 PM	99.9	65	3.61	2.35	50	—	—	—	—	3.73	—	2.48	0.31	VA-ECMO and IABP insertion
5.00 PM	100	81	6.63	4.32	63	8	20	105	663.9	2.15	VA-ECMO and IABP	0.15	0.31	Atrial septostomy
10.00 PM	100	72	5.25	3.42	62	8	20	99	747.1	0.88	VA-ECMO and IABP	0.09	0.31	
Second day														
6.00 AM	100	74	5.9	3.84	65	10	30	100	746.2	1	VA-ECMO and IABP	0.31	0.31	
Fifth day														
6.00 AM	99.9	78	7.26	5	78	11	15	87	738.6	1	VA-ECMO and IABP	0.18	Discontinued	
10th day														
6.00 AM	99.9	75.6	6.62	4.5	86	11	16	71	906.1	1.1	VA-ECMO and IABP	0.18		BiVAD insertion
2.00 PM	100	62	3.3	2.2	71	14	29	103	1379.9	1.32	BiVAD	0.22		
6.00 PM	100	62	3.3	2.2	73	15	24	108	1404.1	1.22	BiVAD	0.22		
10.00 PM	100	62	3.3	2.2	75	15	30	104	1452.5	0.99	BiVAD	0.22		
11th day														
6.00 AM	99.3	59.1	3.52	2.36	74	16	30	114	1316.9	1	BiVAD	0.22		
12th day														
6.00 AM	99	58	3.99	2.67	63	14	41	122	982.6	0.87	BiVAD	0.24		Fibreoptic bronchoscopy
14th day														
2.00 AM	99	54	3.43	2.27	70	15	—	—	1283.3	0.76	BiVAD	0.28		
16th day														
9.00 PM	99	54	3.43	2.27	65	14	—	—	1189.9	—	BiVAD	0.22		
17th day														
9.00 AM	99	66	4.85	3.32	80	15	27	101	1071.2	1.6	BiVAD	0.16		
20th day														
9.00 AM	99.7	70.8	5.29	3.66	59	13	20	76	696.3	1.2	BiVAD	0.05		Orthotopic heart transplant

Abbreviations: BiVAD, biventricular assist device; CI, cardiac index; CO, cardiac output; CVP, central venous pressure; HR, heart rate; IABP, intraaortic balloon pump; MAP, mean arterial pressure; MCS, mechanical circulatory support; PCWP, pulmonary capillary wedge pressure; SaO2, arterial oxygen saturation; SvO2, mixed venous oxygen saturation; SVR, systemic vascular resistance; VA-ECMO, venoarterial extracorporeal membrane oxygenation.

## Data Availability

The data used to support the findings of this study are available from the corresponding author upon request.

## Nomenclature

CCU: cardiac care unit
IPA: invasive pulmonary aspergillosis
VA-ECMO: venoarterial extracorporeal membrane oxygenation
IABP: intraaortic balloon pump
BiVAD: biventricular assist device
BAL: bronchoalveolar lavage
CT: computed tomography
VAP: ventilator-associated pneumonia
RLL: right lower lobe
